# Research on distributionally robust energy storage capacity allocation for output fluctuations in high permeability wind and solar distribution networks

**DOI:** 10.1371/journal.pone.0299226

**Published:** 2024-03-19

**Authors:** Xin Wang, Bo Sun, Cheng Ge, Qian Liu, Zhiwei Li, Mengqi Huang

**Affiliations:** State Grid Anhui Economic Research Institute, Hefei, China; Wuhan University, CHINA

## Abstract

This paper presents a novel approach to addressing the challenges associated with energy storage capacity allocation in high-permeability wind and solar distribution networks. The proposed method is a two-phase distributed robust energy storage capacity allocation method, which aims to regulate the stochasticity and volatility of net energy output. Firstly, an energy storage capacity allocation model is established, which considers energy storage’s investment and operation costs to minimize the total cost. Then, a two-stage distributed robust energy storage capacity allocation model is established with the confidence set of uncertainty probability distribution constrained by 1-norm and ∞-norm. Finally, a Column and Constraint Generation (C&CG) algorithm is used to solve the problem. The validity of the proposed energy storage capacity allocation model is confirmed by examining different wind and solar penetration levels. Furthermore, the model’s superiority is demonstrated by comparing it with deterministic and robust models.

## Introduction

The variability of wind and solar power output in high-permeability wind and solar power distribution networks presents significant challenges to power systems secure and stable operation [1, 2]. Energy storage technology can mitigate energy fluctuations [3, 4], attain stable electricity output, enhance energy management, and optimize energy utilization rates. Thus, energy storage has and become a crucial buffer within the entire distribution network.

The energy storage capacity design will directly impact the distribution network’s reliability and economy. A substantial proposed capacity will escalate initial investment costs and demand more installation space. Insufficient configured capacity can impede efficient storage of distributed energy sources, like photo-voltaic and wind power. This situation results in the waste of solar and wind energy resources and can also adversely impact the safety of the power supply system. Consequently, scientists have widely addressed the matter of energy storage capacity design in distribution networks [5–7].

Reference [8]presented a range of methods for configuring energy storage capacity that incorporates electricity, heat, and exhaust gas. The study analyzed typical daily winter data in isolated mode and compared the energy storage capacity allocation outcomes, leading to enhanced stability of the microgrid system. Reference [9] Performance and cost-related issues of batteries and supercapacitors were analyzed, and a plan for distributing energy storage power by capacity was developed to reduce costs, improve system reliability and efficiency, and extend the life of the equipment. Reference [10] examined the system’s costs under distinct constraints and applied differential algorithms to solve the model. This approach can boost the economy and reliability of the isolated island system. Reference [11] optimized the model for storing wind and solar energy in order to reduce the cost of leveled energy. The model was solved using a Monte Carlo simulation with an embedded gravity search method, resulting in an optimal configuration plan for wind and solar energy storage capacity. Reference [12] presented a methodology for power allocation that uses second-order moving average filtering and a configuration process based on continuous motion to optimize capacity. This approach was applied to hybrid energy storage systems incorporating compressed air, lithium batteries, and supercapacitors. Reference [13] developed a novel model for optimizing energy storage configuration and presented power and capacity assignment techniques for electrical and thermal energy systems. Reference [14] formulated a multi-objective function that considers the objective functions of the investment cost, wind speed, and electricity generation volume. The NSGA-II algorithm was employed to identify the optimal solution set, which enhances energy utilization efficiency and power quality. Reference [15] In this paper, a multi-objective function is proposed to calculate the annual average comprehensive cost under different self-balancing rates by using NSGA-II algorithm and Monte Carlo simulation with annual comprehensive cost and self-balancing function. Reference [16] developed a capacity allocation model for microgrid systems using the minimum total net value and renewable energy utilization rate as evaluation indicators. This model enhances both the economic and environmental benefits. Reference [17] developed a composite model for assessing the total life cycle cost of energy storage in the economical operation of microgrids. The study proposes an integrated configuration approach for planning and operation, which enhances economic benefits.

However, research on energy storage configuration currently focuses on a penetration rate of around 30% or lower for wind and solar power, with minimal attention given to energy storage configuration in high-penetration wind and solar power distribution networks.

Furthermore, the variability and unpredictability of wind and solar energy production present significant obstacles to the secure functioning of the power network, with growing awareness surrounding the uncertainty of renewable energy. Uncertainty research in microgrids mainly encompasses stochastic programming, robust optimization, and distributionally robust optimization methods. The random programming method is a common approach for studying uncertain optimization problems built on the foundation of probability statistics. Reference [18] used a normal distribution to represent the probability density function of wind speed deviation and light intensity prediction deviation. Additionally, the relationship between power generation, wind speed or light intensity was harnessed to establish a joint probability density function of wind power and photovoltaics. Reference [19] explained the volatility bias of renewable energy using the stochastic programming method and developed a two-stage stochastic programming approach. A robust optimization is a valuable approach for analyzing problems in optimization that contain uncertainties. Compared to stochastic programming, robust optimization accounts for errors within a predetermined range, making it easier to manage practical issues in large and medium-sized power systems. Reference [20] considered solar radiation intensity an uncertain parameter in photovoltaic power generation. An uncertainty set was constructed for photovoltaic power generation using a box uncertainty set to address this. Subsequently, a robust optimization model for the ultimate capacity of photovoltaic power plants was established. Reference [21] considered wind speed as a random variable affecting the output of wind turbines. They used box sets to represent wind speed uncertainty and developed a robust optimization model for determining the maximum installed capacity of wind farms. The distributionally robust method, an improved approach based on stochastic programming, combines the benefits of robust optimization for dealing with uncertainty. Reference [22] outlines the range of fluctuations for wind power variables through interval ranges and constructs a fuzzy set of random variables. A two-stage distributionally robust model, under the non-cooperative game mode, is proposed to address the uncertainty of wind power output using the constructed fuzzy set. Reference [23] used the Wasserstein metric to quantify the distinctions between simulated and actual data and developed a distributionally robust multi-level stochastic power flow problem across multiple stages. Building upon historical data, Reference [24] constructed a fuzzy set to account for uncertain loads and renewable energy generation prediction errors and suggested a data-driven distributionally robust approach. Currently, very few studies have employed distributionally robust optimization techniques to address the issue of energy storage configuration with high permeability in wind and solar power distribution networks.

This paper proposes a two-stage energy storage capacity configuration model based on research and analysis. The innovation of the model lies in its distributed robustness, which is suitable for high-permeability wind and solar power distribution networks. The first stage determines the energy storage capacity in high permeability wind and solar power distribution networks, and the second stage simulates the system’s operation after it is put into operation. To address the uncertainty issue of wind and solar energy output, a distributed robust method is adopted, and the confidence set of the uncertainty probability distribution is constrained by combining 1-norm and ∞—norm. Finally, the effectiveness of the proposed model in energy storage capacity allocation under different wind and solar penetration rates was verified through corresponding examples, and the superiority of the model demonstrated through comparison with deterministic and robust models.

## 2. Energy storage capacity configuration model

### 2.1. Objective function

**Fig 1:**
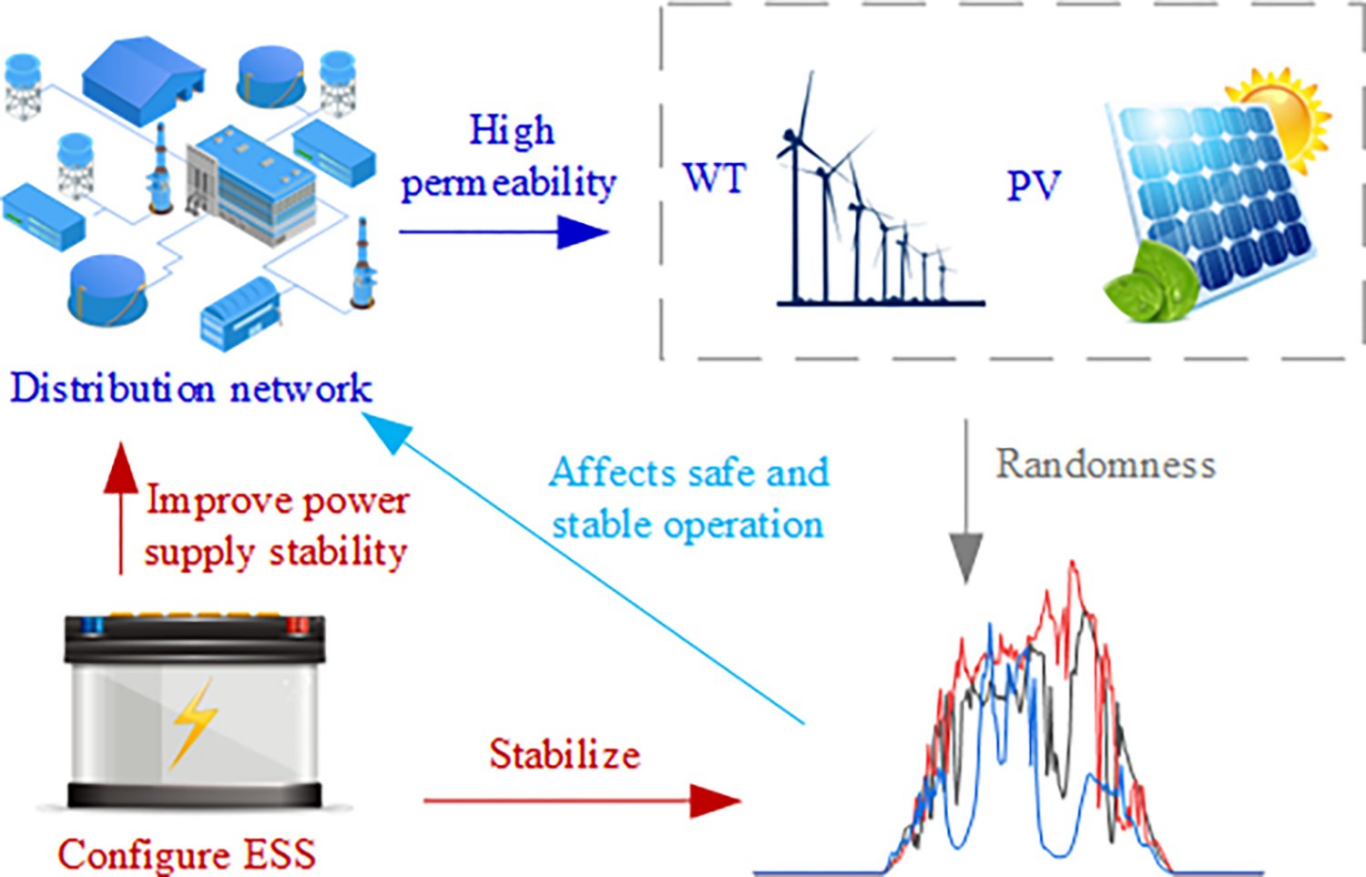
ESS capacity configuration structure block diagram.

This article presents energy storage as a means to reduce the impact of wind and solar uncertainty on the distribution network and finalize the energy storage capacity configuration for high-permeability wind and solar distribution networks. The proposed energy storage capacity configuration model aims to minimize the total cost (including configuration and operation costs). Fig 1 illustrates the structural diagram of the energy storage capacity configuration.

The specific expression of the model objective function is:

$$\min(F^{C,1}+F^{C,2})$$ (1)

$$F^{C,1}=\frac{r_{\mathrm{ESS}}{\left(1+r_{\mathrm{ESS}}\right)}^{y_{\mathrm{ESS}}}}{{\left(1+r_{\mathrm{ESS}}\right)}^{y_{\mathrm{ESS}}}-1}\sum_{j\in B_{\mathrm{ESS}}}\beta_{\mathrm{con}}^{\mathrm{ESS}}S_{j}^{\mathrm{ESS}}$$ (2)

$$F^{C,2}=F^{\mathrm{Sub}}+F^{\mathrm{PV}}+F^{\mathrm{WT}}+F^{\mathrm{ESS}}+F^{\mathrm{Loss}}$$ (3)

$$F^{\mathrm{Sub}}=\sum_{t=1}^{T}\sum_{j\in B_{\mathrm{sub}}}c_{t}^{\mathrm{sub}}P_{j,t}^{\mathrm{sub}}$$ (4)

$$F^{\mathrm{PV}}=\sum_{t=1}^{T}\sum_{j\in B_{\mathrm{PV}}}c_{t}^{\mathrm{PV}}P_{j,t,cur}^{\mathrm{PV}}$$ (5)

$$F^{\mathrm{WT}}=\sum_{t=1}^{T}\sum_{j\in B_{\mathrm{WT}}}c_{t}^{\mathrm{WT}}P_{j,t,cur}^{\mathrm{WT}}$$ (6)

$$F^{\mathrm{ESS}}=\sum_{t=1}^{T}\sum_{j\in B_{\mathrm{ESS}}}c_{t}^{\mathrm{ESS}}(P_{j,t}^{\mathrm{ch}}+P_{j,t}^{\mathrm{dch}})$$ (7)

$$F^{\mathrm{Loss}}=\sum_{t=1}^{T}\sum_{l\in B_{l}}c_{t}^{\mathrm{loss}}I_{l,t}^{2}R_{l}$$ (8)

Where: $\beta_{\mathrm{con}}^{\mathrm{ESS}}$ represents the construction cost per unit capacity of energy storage; *r*_ESS_ and *y*_ESS_ describe the discount rate of energy storage and the total life cycle of the equipment; $S_{j}^{\mathrm{ESS}}$ is the energy storage access capacity; *F*^Sub^, *F*^PV^, *F*^WT^, *F*^ESS^, and *F*^Loss^ respectively represent the cost of purchasing electricity from the superior power grid, the penalty for light abandonment, the penalty for light abandonment, the aging cost of energy storage operation, and the cost of network loss; *B*_sub_, *B*_PV_, *B*_WT_, *B*_ESS_, and *B*_*l*_ are respectively the set of substation nodes, the set of photovoltaic candidate access nodes, the set of wind turbine candidate access nodes, the set of energy storage access nodes, and the set of lines; $c_{t}^{\mathrm{sub}}$, $c_{t}^{\mathrm{PV}}$, $c_{t}^{\mathrm{WT}}$, $c_{t}^{\mathrm{ESS}}$, and $c_{t}^{\mathrm{loss}}$ are the unit prices for purchasing electricity, discarding solar energy, discarding wind, aging energy storage, and network loss from the superior power grid; $P_{j,t}^{\mathrm{sub}}$, $P_{j,t,cur}^{\mathrm{PV}}$, $P_{j,t,cur}^{\mathrm{WT}}$, $P_{j,t}^{\mathrm{ch}}$, and $P_{j,t}^{\mathrm{dch}}$ are the active output, photovoltaic power, wind turbine power, energy storage charging power, and energy storage discharge power of the substation; *I*_*l*,*t*_ and *R*_*l*_ respectively represent the current and resistance of branch *l*.

### 2.2. Constraint condition

#### 2.2.1. Energy storage capacity configuration constraints

For distribution network operators, there is an upper limit value for investment in energy storage, expressed as

$$F^{C,1}\leq F^{\mathrm{Con},\max}$$ (9)

Where: *F*^Con, max^ represents the maximum investment budget for energy storage.

In addition, adding the maximum investment constraint can also avoid the model being unsolvable.


$$\sum_{l(j,:)\in B_{l}}P_{l,t}=\sum_{l(:,j)\in B_{l}}\left(P_{l,t}-I_{l,t}^{2}R_{l}\right)-P_{j,t}$$ (10)

$$\sum_{l(j,:)\in B_{l}}Q_{l,t}=\sum_{l(:,j)\in B_{l}}\left(Q_{l,t}-I_{l,t}^{2}X_{l}\right)-Q_{j,t}$$ (11)

$$P_{j,t}=P_{j,t}^{\mathrm{load}}-P_{j,t}^{\mathrm{sub}}-P_{j,t}^{\mathrm{WT}}-P_{j,t}^{\mathrm{PV}}-P_{j,t}^{\mathrm{ch}}+P_{j,t}^{\mathrm{dch}}$$ (12)

$$Q_{j,t}=Q_{j,t}^{\mathrm{load}}-Q_{j,t}^{\mathrm{sub}}-Q_{j,t}^{\mathrm{WT}}$$ (13)

$$U_{j,t}^{2}=U_{i,t}^{2}-2(P_{l,t}R_{l}+Q_{l,t}X_{l})+I_{l,t}^{2}(R_{l}^{2}+X_{l}^{2})$$ (14)

$$I_{l,t}^{2}U_{i,t}^{2}=P_{l,t}^{2}+Q_{l,t}^{2}$$ (15)

#### 2.2.2. AC power flow constraints

Where: *l*(*j*,:) represents a branch with node *j* as the root node; *l*(:,*j*) represents a branch with node *j* as its child node; *X*_*l*_ is the reactance of branch *l*; *i* and *j* are the starting and ending points of the branch road, and *Q*_*l*,*t*_ are the active and reactive power of branch *l*; $P_{j,t}^{\mathrm{load}}$, $P_{j,t}^{\mathrm{PV}}$, and $P_{j,t}^{\mathrm{WT}}$ are the dynamic load, photovoltaic active output, and wind turbine active output; $Q_{j,t}^{\mathrm{load}}$, $Q_{j,t}^{\mathrm{sub}}$, and $Q_{j,t}^{\mathrm{WT}}$ are the reactive loads, substation reactive power, and wind turbine reactive output.

#### 2.2.3. Safety operation constraints


$$\left\{ \begin{array}{c} P_{l,t}^{2}+Q_{l,t}^{2}\leq S_{l,\max}^{2}\\ P_{l,t}\leq S_{l,\max} \end{array}\right.$$ (16)

$$\left\{ \begin{array}{c} U_{\min}\leq U_{j,t}\leq U_{\max}\\ 0\leq I_{l,t}\leq I_{\max} \end{array}\right.$$ (17)

Where: *S*_*l*,max_ is the upper limit of the current carrying capacity; *U*_max_ and *U*_min_ are the upper and lower limits of the node voltage; *I*_max_ is the upper current limit.

#### 2.2.4. Substation power constraints


$$\left\{ \begin{array}{c} P_{\mathrm{sub}}^{\min}\leq P_{j,t}^{\mathrm{sub}}\leq P_{\mathrm{sub}}^{\max}\\ Q_{\mathrm{sub}}^{\min}\leq Q_{j,t}^{\mathrm{sub}}\leq Q_{\mathrm{sub}}^{\max} \end{array},j\in B_{\mathrm{sub}}\right.$$ (18)

Where: $P_{\mathrm{sub}}^{\max}$ and $P_{\mathrm{sub}}^{\min}$, $Q_{\mathrm{sub}}^{\max}$ and $Q_{\mathrm{sub}}^{\min}$ are the upper and lower limits of the substation nodes’ active and reactive power output, respectively.

#### 2.2.5. Distributed new energy constraints

1) Photovoltaic unit(PV) constraints

$$\left\{ \begin{array}{c} P_{j,t}^{\mathrm{PV}}+P_{j,t,cur}^{\mathrm{PV}}=P_{j,t,\max}^{\mathrm{PV}}\\ P_{j,t}^{\mathrm{PV}},P_{j,t,cur}^{\mathrm{PV}}\geq 0,P_{j,t,\max}^{\mathrm{PV}}\leq S_{j}^{\mathrm{PV}} \end{array},j\in B_{\mathrm{PV}}\right.$$ (19)

Where: $P_{j,t,\max}^{\mathrm{PV}}$ is the maximum output of photovoltaic active power.

2) Wind turbine(WT) constraints

$$\left\{ \begin{array}{c} P_{j,t}^{\mathrm{WT}}+P_{j,t,cur}^{\mathrm{WT}}=P_{j,t,\max}^{\mathrm{WT}}\\ P_{j,t}^{\mathrm{WT}},P_{j,t,cur}^{\mathrm{WT}}\geq 0,P_{j,t,\max}^{\mathrm{WT}}\leq S_{j}^{\mathrm{WT}}\\ Q_{j,t}^{\mathrm{WT}}=\tan(\cos^{-1}\varphi^{\mathrm{WT}})P_{j,t}^{\mathrm{WT}} \end{array},j\in B_{\mathrm{WT}}\right.$$ (20)

Where: $P_{j,t,\max}^{\mathrm{WT}}$ is the maximum active output of the WT; *φ*^WT^ is the power factor of the wind turbine unit.

#### 2.2.6. Energy storage system (ESS) constraints


$$E_{j,t+1}=E_{j,t}+\eta_{ch,t}P_{j,t}^{\mathrm{ch}}\Delta t-P_{j,t}^{\mathrm{dch}}⁄\eta_{dch,j}\Delta t$$ (21)

$$E_{j,\max}\times 20\%\leq E_{j,t}\leq E_{j,\max}\times 90\%$$ (22)

$$M_{\mathrm{ch},j,t}+M_{\mathrm{dch},j,t}\leq 1$$ (23)

$$\left\{ \begin{array}{c} 0\leq P_{j,t}^{\mathrm{ch}}\leq P_{\mathrm{ch},j,\max}M_{\mathrm{ch},j,t}\\ 0\leq P_{j,t}^{\mathrm{dch}}\leq P_{\mathrm{dch},j,\max}M_{\mathrm{dch},j,t} \end{array}\right.$$ (24)

Where: *E*_*j*,*t*_ is the electricity level in ESS; *η*_*ch*,*t*_ and *η*_*dch*,*j*_ respectively measure the charging and discharging efficiency of the ESS; *E*_*j*,max_ is the maximum capacity of ESS; *M*_ch,*j*,*t*_ and *M*_dch,*j*,*t*_ are variables of 0 and 1, where 1 represents charging (discharge) and 0 represents not charging (discharge), ensuring that charging and discharging cannot occur simultaneously; *P*_ch,*j*,max_ and *P*_dch,*j*,max_ are the maximum power for ESS charging and discharging, respectively; *B*_ESS_ is the set of ESS access nodes.

### 2.3. Distributionally robust energy storage capacity allocation model

To reduce the effects of widespread wind and solar energy on the distribution grid, this study recommends a two-phase energy storage capacity design that is distributionally robust. The initial stage determines the capacity of ESS access, while the second stage is focused on operation. After establishing the first-stage setting, a simulation process is carried out to determine the lowest operating cost. For ease of representation, ***x*** it denotes the first stage variable, which comprises the capacity of energy storage configuration. Meanwhile, the second stage variable, represented by ***y***_*s*_, encompasses operating variables like the main network’s electricity purchase quantity and energy storage power discharge and charging. Thus, the two-stage distributionally robust model for energy storage capacity configuration is expressed as follows:

$$\underset{x\in X}{\min}Ax+\underset{p_{s}\in \Omega^{p}}{\max}\sum_{s=1}^{N_{s}}p_{s}\underset{y_{s}\in Y}{\min}By_{s}$$ (25)

s.t.Cx≤c (26)

$$\left\{ \begin{array}{c} Ey_{s}\leq e\\ Fy_{s}=f \end{array},\;\forall s\right.$$ (27)

$$\left\{ \begin{array}{c} Gx+Hy_{s}\leq g\\ Kx+My_{s}=u \end{array},\;\forall s\right.$$ (28)

$$\|Qy_{s}+P{\|}_{2}\leq q^{T}y_{s}+p,\;\forall s$$ (29)

Where: Ω^*p*^ is the set that *p*_*s*_ satisfies; *N*_*s*_ represents the total number of scenes; ***A*** represents the configuration layer coefficient matrix; ***B*** represents the coefficient matrix of the operating layer; ***Ax*** in objective function Eq (25) represents the configuration cost "*F*^C,1^"; ***By***_*s*_ represents the operating cost "*F*^C,2^" in scenario *s*; Eq (26) represents the first stage variable related constraints, corresponding precisely to Eq (9); Eq (27) represents the second stage variable related constraints, corresponding to Eqs (16) to (24); Eq (28) represents the coupling constraint relationship between variables in the first and second stages, corresponding to Eqs (10) to (15); Eq (29) is the corresponding Eq (40) for the second-order cone relaxation constraint of the power flow.

## 3. Handling the uncertainty

Considering the uncertainty of the high permeability landscape, a theoretical distribution $p_{s}^{0}$ containing uncertain variables is constructed based on historical data, and a confidence set is established to achieve the actual distribution, represented as:

$$\Omega^{p}=\{\left\{p_{s}\}|\begin{array}{c} p_{s}\geq 0,s=1,\ldots ,N_{s}\\ \sum_{s=1}^{N_{s}}p_{s}=1\\ \sum_{s=1}^{N_{s}}|p_{s}-p_{s}^{0}|\leq \theta_{1}\\ \underset{1\leq s\leq N_{s}}{\max}|p_{s}-p_{s}^{0}|\leq \theta_{\infty } \end{array}\right\}$$ (30)

Where: *θ*_1_ and *θ*_∞_ are the allowable deviation limits for probabilities under 1-norm and ∞-norm corresponding constraints.

The confidence set can be constructed based on the confidence level and the number of historical data, expressed as:

{Pr{∑s=1Ns|ps−ps0|≤θ1}≥1−2Nse−2Mθ1NsPr{max1≤s≤Ns|ps−ps0|≤θ∞}≥1−2Nse−2Mθ∞ (31)

Order

{α1=1−2Nse−2Mθ1Nsα∞=1−2Nse−2Mθ∞ (32)

There are:

$$\left\{ \begin{array}{l} \theta_{1}=\frac{N_{s}}{2M}\ln\frac{2N_{s}}{1-\alpha_{1}}\\ \theta_{\infty }=\frac{1}{2M}\ln\frac{2N_{s}}{1-\alpha_{\infty }} \end{array}\right.$$ (33)

Where: *M* represents the number of historical data; *α*_1_ and *α*_∞_ are uncertainty probability confidence levels.

Based on Eqs (30) to (33), the uncertainty of wind and solar energy is processed to obtain a distributionally robust energy storage capacity configuration model, represented as:

$$\left\{ \begin{array}{c} \underset{x\in X}{\min}Ax+\underset{p_{s}\in \Omega^{p}}{\max}\sum_{s=1}^{N_{s}}p_{s}\underset{y_{s}\in Y}{\min}By_{s}\\ s.t.(30)‐(33) \end{array}\right.$$ (34)

## 4. Model solution

### 4.1. Model equivalence processing

#### 4.1.1. Second-order cone relaxation under tidal current constraints

If Eq (35) is used to replace the square terms of current and voltage in the power flow constraints Eqs (10) to (11) and (14) to (15), the corresponding constraints will be converted to Eqs (36) to (39).


$$\left\{ \begin{array}{c} I_{l,t}^{\varphi }=I_{l,t}^{2}\\ V_{i,t}^{\varphi }=V_{i,t}^{2} \end{array}\right.$$ (35)

$$\sum_{l(j,:)\in B_{l}}P_{l,t}=\sum_{l(:,j)\in B_{l}}\left(P_{l,t}-I_{l,t}^{\varphi }R_{l}\right)-P_{j,t}$$ (36)

$$\sum_{l(j,:)\in B_{l}}Q_{l,t}=\sum_{l(:,j)\in B_{l}}\left(Q_{l,t}-I_{l,t}^{\varphi }X_{l}\right)-Q_{j,t}$$ (37)

$$V_{j,t}^{\varphi }=V_{i,t}^{\varphi }-2(P_{l,t}R_{l}+Q_{l,t}X_{l})+I_{l,t}^{\varphi }(R_{l}^{2}+X_{l}^{2})$$ (38)

$$I_{l,t}^{\varphi }V_{i,t}^{\varphi }=P_{l,t}^{2}+Q_{l,t}^{2}$$ (39)

Perform second-order cone relaxation on Eq (39), and the corresponding constraints are transformed into:

$${\left\|\begin{array}{c} 2P_{l,t}\\ 2Q_{l,t}\\ I_{l,t}^{\varphi }-V_{i,t}^{\varphi } \end{array}\right\|}_{2}\leq I_{l,t}^{\varphi }+V_{i,t}^{\varphi }$$ (40)

#### 4.1.2. Absolute value-constrained linearization

The constraint in Eq (30) exists as an absolute value constraint, and the auxiliary variable *k* is introduced for equivalent transformation.

The *θ*_1_ constraints after conversion are as follows:

$$\sum_{s=1}^{N_{s}}k\leq \theta_{1}$$ (41)

$$\{\begin{array}{l} k\geq p_{s}-p_{s}^{0}\\ k\geq p_{s}^{0}-p_{s} \end{array},\;\forall s$$ (42)

The *θ*_∞_ constraints after conversion are as follows:

$$\{\begin{array}{l} p_{s}-p_{s}^{0}\leq \theta_{\infty }\\ p_{s}^{0}-p_{s}\leq \theta_{\infty } \end{array},\;\forall s$$ (43)

### 4.2. Distributionally robust model solution

For the two-stage distributionally robust energy storage capacity configuration model established in this article, C&CG decomposes the model into the master problem (MP) and subproblem (SP) for the iterative solution.

The main problem is shown in Eq (44), where the optimal solution that satisfying the constraint is solved under a known probability distribution, and the lower bound value is provided for Eq (25):

$$\mathrm{MP}:\underset{x\in X,y_{s}^{m}\in Y,\eta }{\min}Ax+\eta$$ (44)

$$s.t.\;\eta \geq \sum_{s=1}^{N_{s}}p_{s}^{m}\underset{y_{s}^{m}\in Y}{\min}By_{s}^{m}\;\forall m=1,2,\cdots ,n$$ (45)

Cx≤c (46)

$$\left\{ \begin{array}{c} Ey_{s}^{m}\leq e\\ Fy_{s}^{m}=f \end{array}\;\forall s,\forall m=1,2,\cdots ,n\right.$$ (47)

$$\left\{ \begin{array}{c} Gx+Hy_{s}^{m}\leq g\\ Kx+My_{s}^{m}=u \end{array}\;\forall s,\forall m=1,2,\cdots ,n\right.$$ (48)

$$\|Qy_{s}+P{\|}_{2}\leq q^{T}y_{s}^{m}+p\;\forall s,\forall m=1,2,\cdots ,n$$ (49)

The subproblem is shown in Eq (50). Given the first stage variable x* in the main problem, search for the worst-case probability distribution, return it to the main problem, and provide the upper bound value for Eq (25):

$$\mathrm{SP}:f_{\mathrm{SP}}(x^{*})=\underset{p_{s}\in \Omega^{p}}{\max}\sum_{s=1}^{N_{s}}p_{s}^{m}\underset{y_{s}^{m}\in Y}{\min}By_{s}^{m}\;\forall m=1,2,\cdots ,n$$ (50)

Due to the independence between the discrete scenario probability values and the second-stage variables in the subproblem, the subproblem can be solved in two steps, first solving the inner minimum value problem and then the outer problem.

Step 1: Set the lower and upper bound to *UB* = ∞, so that the number of iterations is *n* = 1.

Step 2: Solve the main problem (44), obtain the optimal solution (x*,η*), and update the lower bound value *LB* = Ax*+η*.

Step 3: Fix the first stage variable x*, solve subproblem (50) to obtain the worst-case probability and objective function values *f*_SP_(x*), and update the upper bound value min{*UB*,Ax*+*f*_SP_(x*)}.

Step 4: If *UB*−*LB*<*ε*, stop the iteration and return to the optimal solution x*; On the contrary, update the worst scenario probability distribution $p_{s}^{n+1}=p_{s}^{*}$ of the main problem and add new variables $y_{s}^{n+1}$ and constraints related to the new variables in Eqs (46) to (49) to the main problem.

Step 5: Update *n* = *n*+1 and return to step 2.

### 4.3. Model solving process

**Fig 2:**
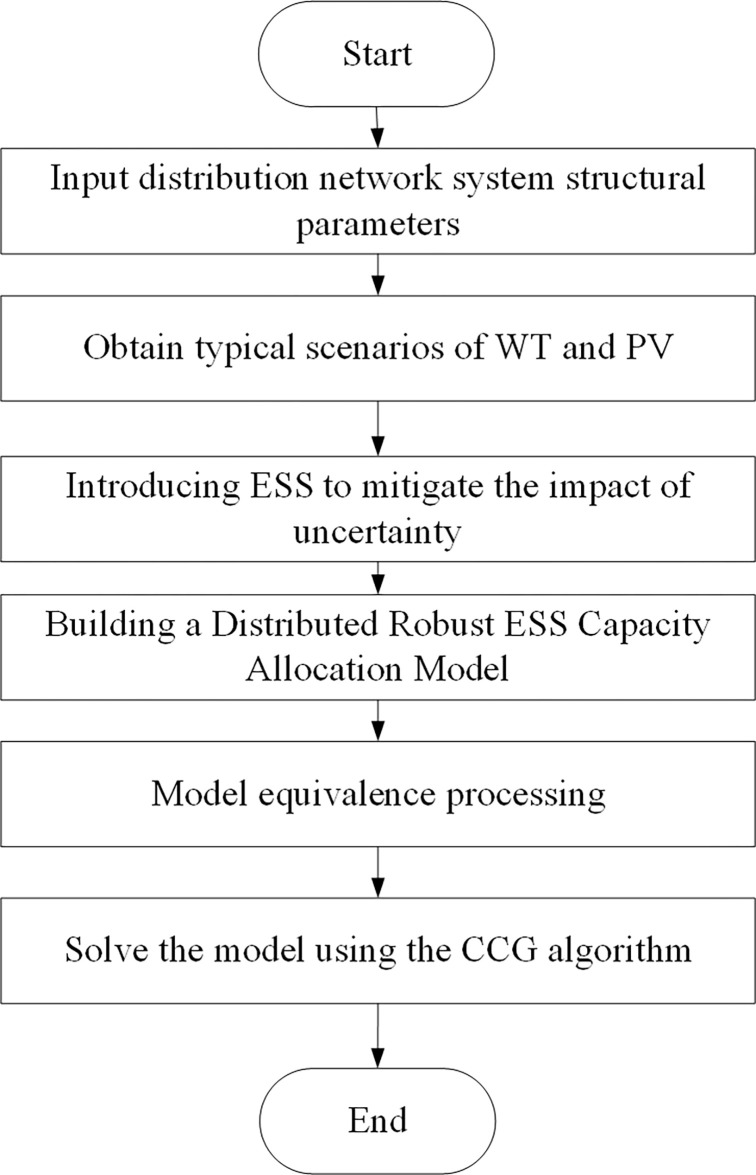
Solving process.

The solution process is shown in Fig 2, which includes parameter input, model establishment, model equivalence processing, and other parts.

## 5. Example analysis

**Fig 3:**
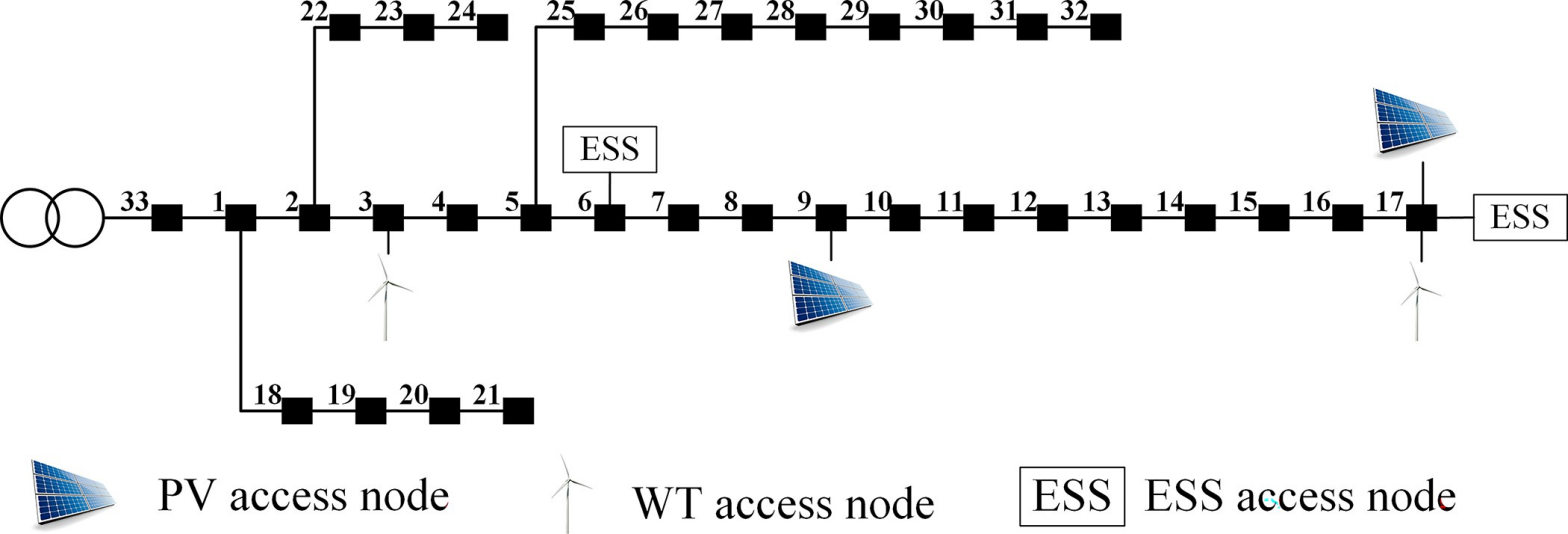
Improved IEEE33 system.

This article takes modifying the IEEE33 node system as an example to verify the effectiveness of the model and algorithm proposed in this paper. The system structure is shown in Fig 3. The system reference values are *S*_base_ = 10MVA and *U*_basc_ = 12.66kV respectively, and other relevant parameters are shown in Table 1 [25].

**Table 1:** Model parameter.

Parameters	Numerical value	Parameters	Numerical value
ESS Life Cycle (Years)	20	Maximum ESS charging and discharging power (MW)	0.3
ESS discount rate	0.08	ESS charging efficiency	0.938 1
ESS unit installation price ($/MW)	240 000	ESS discharge efficiency	0.938 1
ESS Aging Cost ($/MWh)	20	Total active load (MW)	3.715
PV Abandoned photovoltaic Price ($/MWh)	50	Total reactive load (Mvar)	2.3
WT Abandoned Wind Price ($/MWh)	50	Total simulation period (h)	24
Network loss price ($/MWh)	50	Line capacity (MW)	6
Upper voltage limit (p.u.)	1.06^2^	Upper limit of WT power factor	0.9
Lower voltage limit (p.u.)	0.94^2^	Number of historical data	5 000
Substation node	33	1/∞-norm corresponds to uncertainty probability confidence	0.99

**Fig 4:**
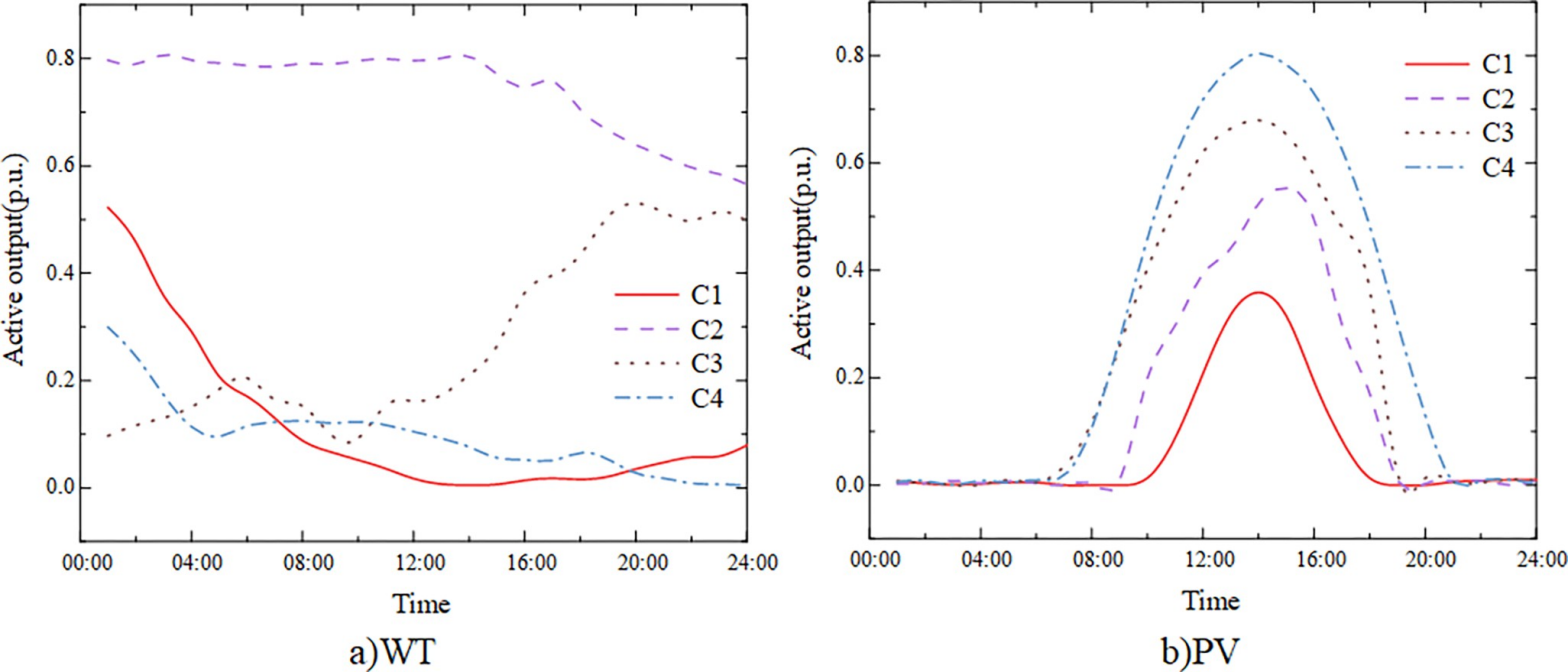
Typical scenarios of WT and PV output.

The system has two access nodes for photovoltaic and wind turbines, located at nodes 9 and 17 for photovoltaic access and nodes 3 and 17 for wind turbine access. The results of wind and solar scenarios are presented in Fig 4, which includes four specific scenarios, designated as C1, C2, C3 and C4, with respective probabilities of 0.227, 0.156, 0.381, and 0.236. The four scenarios are further divided as follows: Scenario 1 has low output of wind and solar power, Scenario 2 is characterized by higher wind power output but lower photovoltaic output, Scenario 3 features higher output from both wind and solar power, and Scenario 4 has lower wind power output but higher output from photovoltaics. In the calculation example, access nodes 9 and 17 are selected to fully set the energy storage capacity. Node 6’s energy storage configuration considers the wind turbines at node 3 and photovoltaic energy at node 9, while node 17 completes the energy storage capacity configuration in the "wind and solar energy integration."

The simulation described in this article utilizes MATLAB R2018a and Gurobi 10.0.3 in order to solve the problem of calculating energy storage capacity. The system’s environment consists of an Intel Core i7-12700 CPU with 16GB of memory.

### 5.1. Analysis of Energy Storage Configuration Capacity under Different Permeability

Firstly, Table 2 analyses the energy storage capacity configuration results under different permeabilities. For clarity, we refer to the wind turbines at Node 3 and Node 17 as WT1 and WT2, respectively, the photovoltaic systems at Node 9 and Node 17 as PV1 and PV2, respectively, and the energy storage systems at Node 6 and Node 17 as ESS1 and ESS2, respectively. All subsequent descriptions employ these abbreviations. At this penetration rate, wind and solar access capacity is further described as the capacity of wind and solar energy that can be accessed.

**Table 2:** ESS configuration capacity under different permeability.

Different permeabilities (%)	Corresponding WT and PV access capacity (MW)	ESS configuration capacity (MWh)
30	WT1(1.01),WT2(0.30) PV1(0.97),PV2(0.23)	ESS1(0.42) ESS2(0.42)
40	WT1(1.34),WT2(0.38) PV1(1.30),PV2(0.31)	ESS1(0.57) ESS2(0.54)
50	WT1(1.68),WT2(0.47) PV1(1.61),PV2(0.37)	ESS1(0.77) ESS2(0.63)
60	WT1(2.02),WT2(0.58) PV1(1.95),PV2(0.46)	ESS1(0.94) ESS2(0.74)
70	WT1(2.34),WT2(0.71) PV1(2.26),PV2(0.57)	ESS1(0.99) ESS2(0.98)

It is clear that this article’s optimization configuration models can reasonably configure energy storage capacity under varying wind and solar energy penetration rates. Additionally, when the penetration rate is 30%, ESS1’s and ESS2’s configured capacity is almost identical at 0.42MWh. However, as the penetration rate rises, ESS1’s energy storage capacity increases more rapidly than ESS2’s. At a wind and solar penetration rate of 60%, the capacity difference between ESS1 and ESS2 reaches its maximum of 0.20 MWh. Subsequently, as the rate of penetration increases, the difference between the two decreases abruptly. At a wind and solar penetration rate of 70%, the difference between ESS1 and ESS2 decreases from the previous maximum of 0.20 MWh to 0.1 MWh.

### 5.2. Analysis of energy storage output in typical scenarios

**Fig 5:**
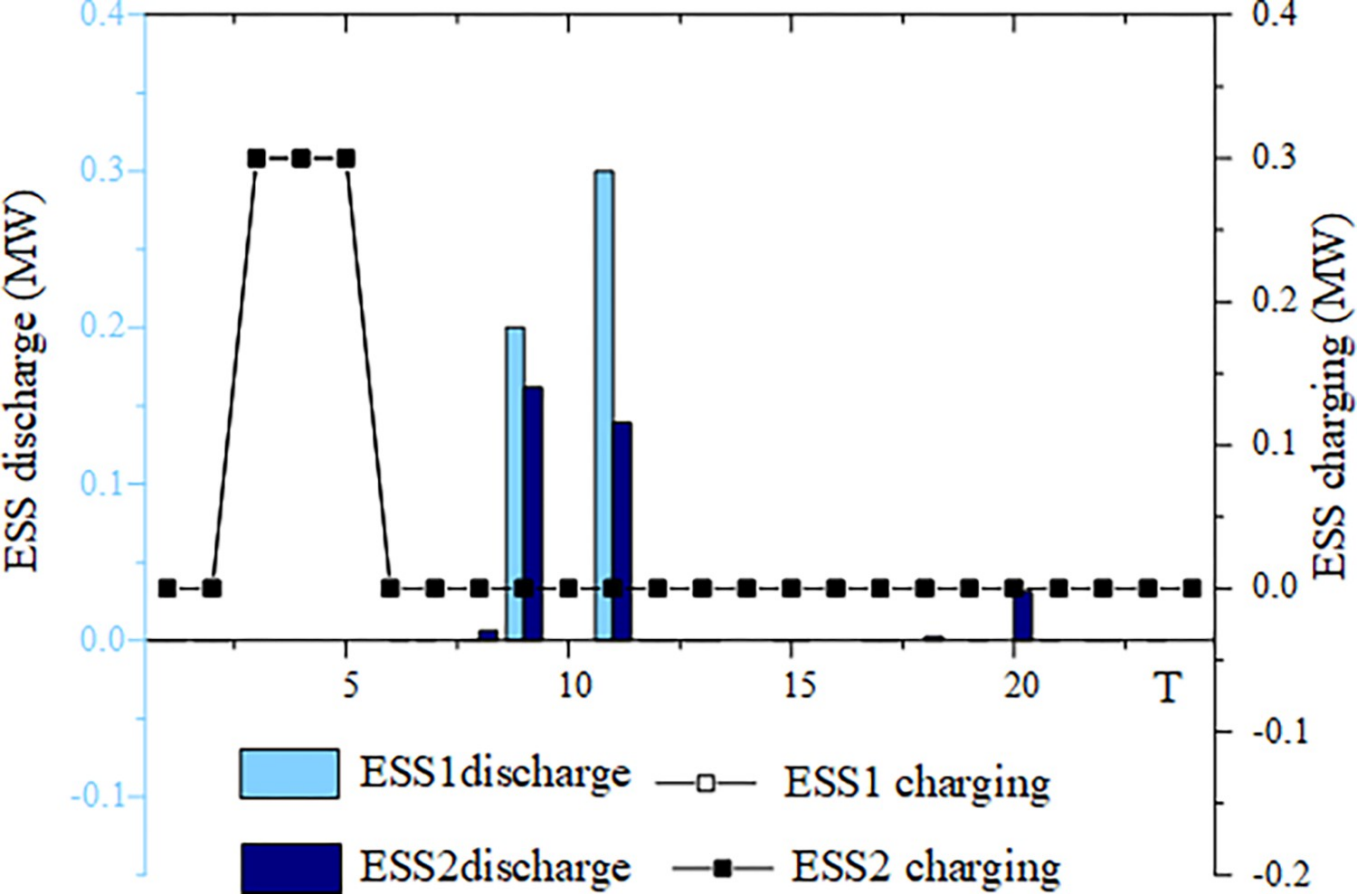
ESS charging and discharging situation in scenario 1.

**Fig 6:**
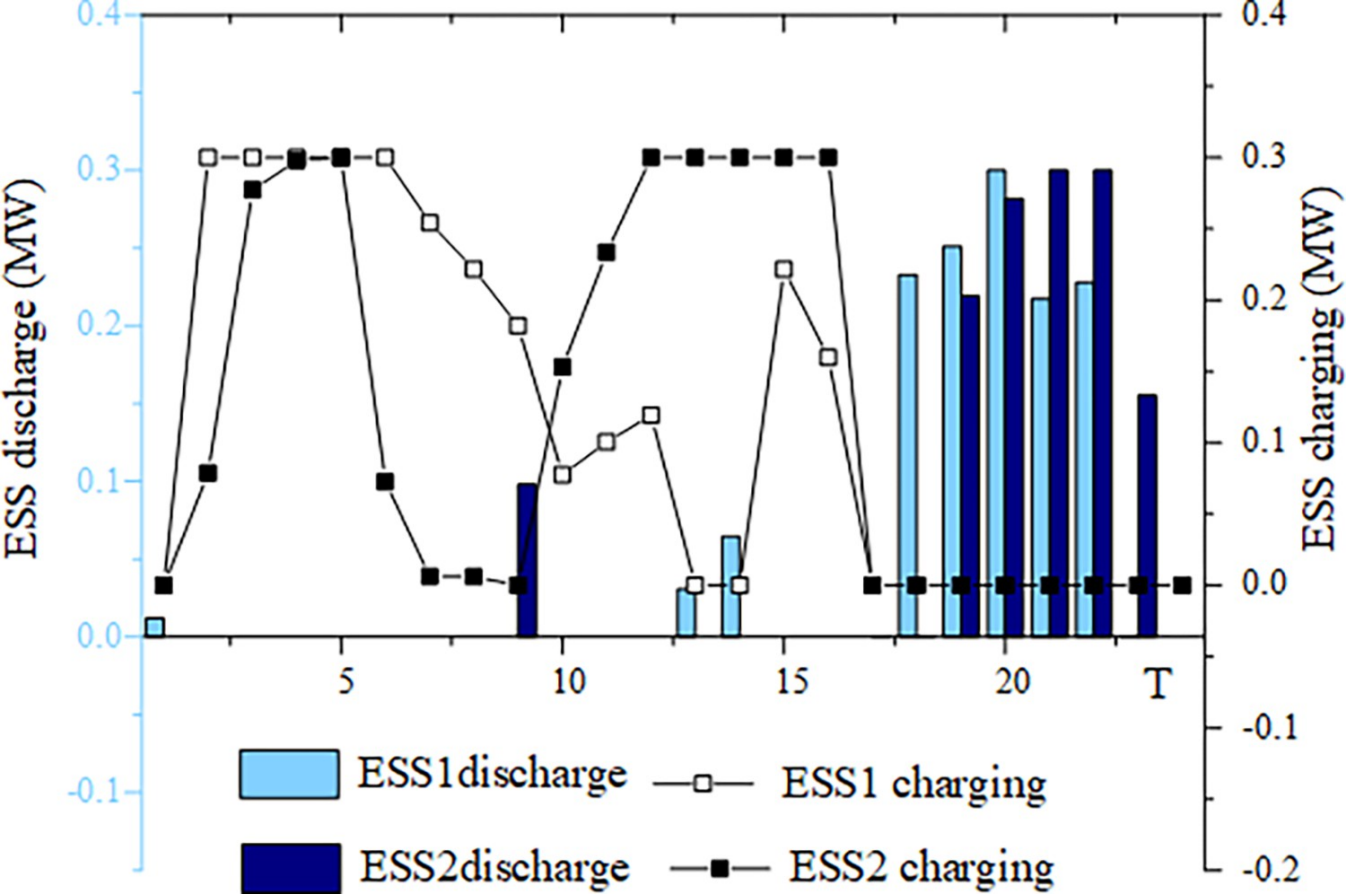
ESS charging and discharging situation in scenario 2.

**Fig 7:**
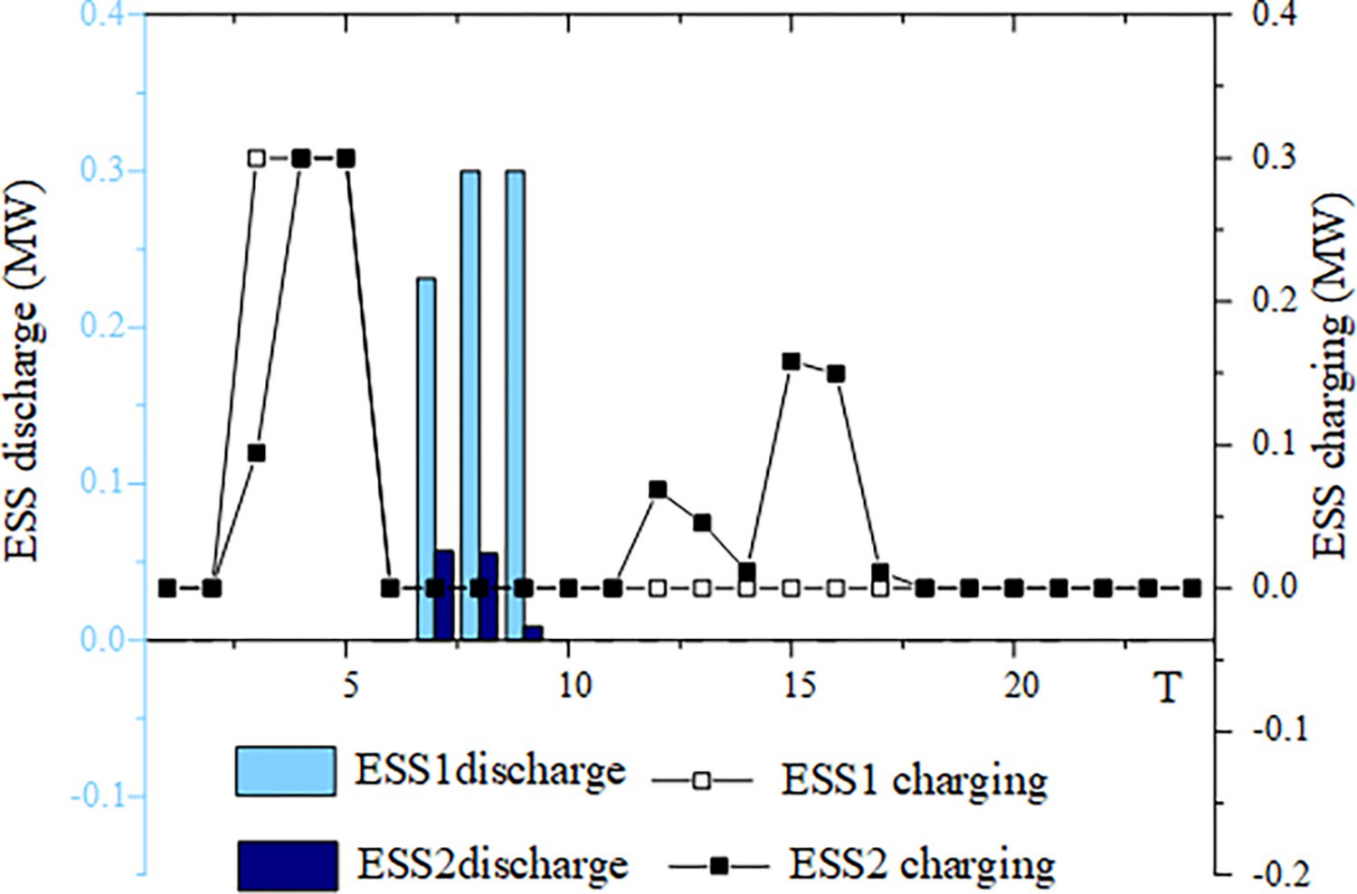
ESS charging and discharging situation in scenario 3.

**Fig 8:**
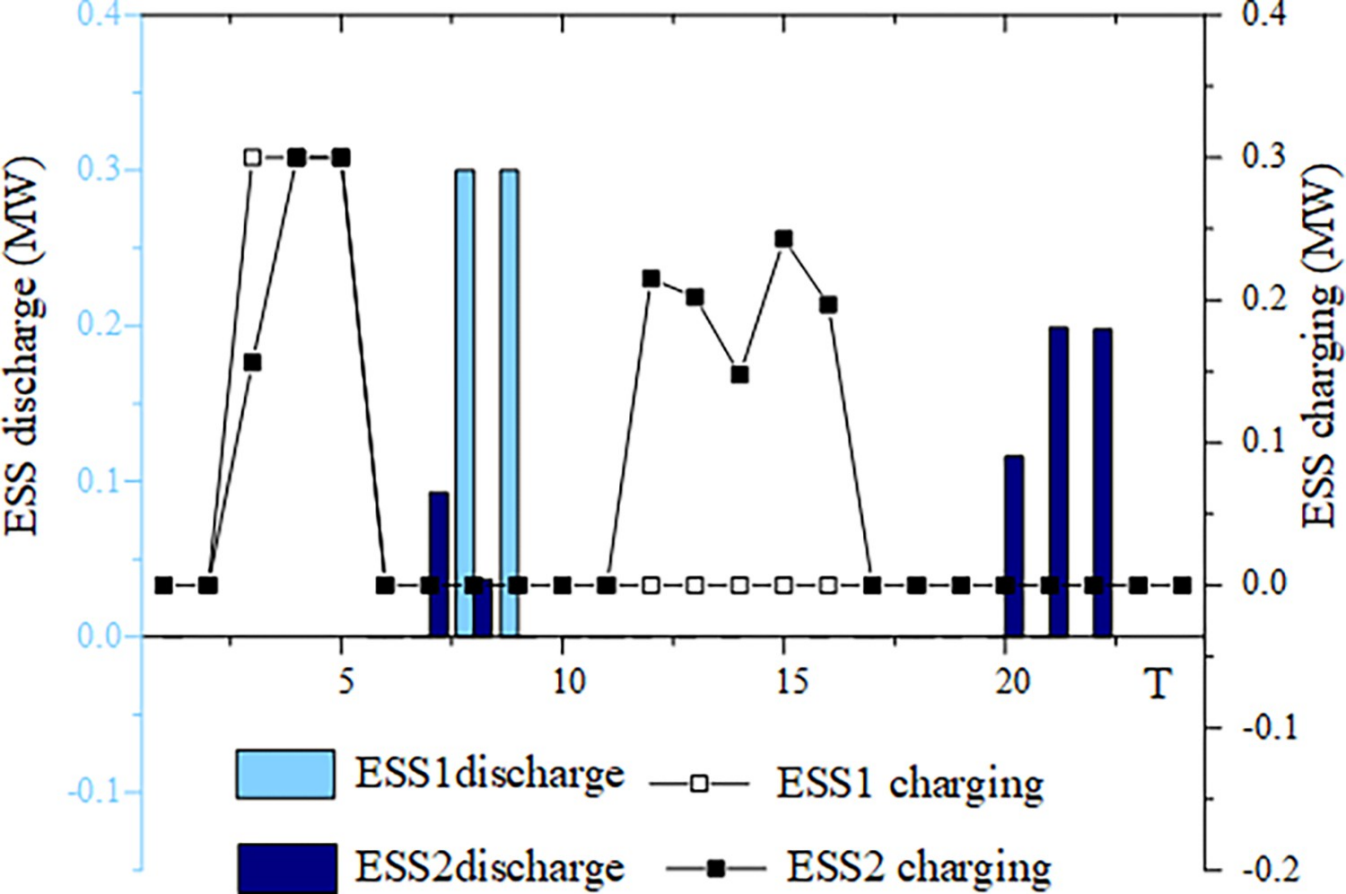
ESS charging and discharging situation in scenario 4.

This section analyzes energy storage charging and discharging under four typical wind and solar energy output scenarios. The example chosen is based on a 50% wind and solar energy penetration rate. Figs 5–8 illustrates the energy storage output in these four scenarios.

For scenario 1, when wind and solar output is low, ESS1 and ESS2 have dedicated charging periods between 3:00 and 5:00. ESS1 discharges at 9:00 and 11:00, while ESS2 discharges at 8:00, 9:00, 11:00, and 20:00. At the “integration of wind and solar energy storage”, the discharge power of ESS1 is higher than that of ESS2. This is especially noticeable at 11:00. At present, energy storage’s involvement in the distribution network’s scheduling primarily concentrates on system stability.

For Scenario 2, characterized by high wind power output and low photovoltaic output, ESS1 charges between 2:00–12:00 and 15:00–16:00. ESS charging, on the other hand, is concentrated between 2:00–8:00 and 10:00–16:00, with only a minor charging power of 0.01MW between 7:00–8:00. The discharge periods for ESS1 are 1:00, 13:00–14:00, and 18:00–22:00, while for ESS2, it is 9:00 and 19:00–23:00. In Scenario 2, the wind turbine output was excessively high before 16:00, and when combined with the active output of the photovoltaic system, the wind, and solar output exceeded the load demand, resulting in both ESS1 and ESS2 charging to consume the excess electricity generated by the wind and solar system. After 4 p.m., the output of the wind turbine decreases, and the photovoltaic output is negligible after 6 p.m. Consequently, the output of the wind turbine is unable to meet the load demand and energy storage is discharged to ensure power supply during this time. As a result, energy storage becomes a key component of system dispatch by absorbing excess power generated by wind and solar, and then utilizes it when the output from these sources is insufficient to meet demand.

For high wind and solar output scenario 3, ESS1 charges primarily between 3:00 and 5:00, while ESS2 charges both between 3:00 and 5:00 and from 12:00 to 17:00. ESS1 discharges between 7:00 and 9:00, and ESS2 discharges during the same period as well. ESS2 is charged more frequently at the "integration of wind and solar energy storage" location in this scenario. However, the discharge periods for ESS1 and ESS2 are comparable, and in contrast to ESS2 at the "integration of wind and solar energy storage" location, ESS1 has a higher discharge power. As a result, energy storage’s involvement in system scheduling is aimed at absorbing excess electricity generated by wind and solar power.

In scenario 4, where there is low wind power output but high photovoltaic output, ESS1 is charged between 3:00 and 5:00 and ESS2 is charged between 3:00 and 5:00, as well as 12:00 to 16:00. ESS1 discharges between 8:00 and 9:00, while ESS2 discharges between 7:00 and 8:00 and again between 20:00 and 22:00. ESS2 has more frequent charging and discharging cycles in the "integration of wind and solar energy storage" sector compared to ESS1. During this phase, energy storage operates in system scheduling to absorb excess electricity from wind and solar power and to provide electricity when the output of wind and solar power is inadequate.

Figs 5–8 highlight the importance of configuring energy storage to maintain power supply balance and stabilize output fluctuations. Energy storage charging involves the storage of excess electricity generated from photovoltaic and wind power, thereby reducing the phenomenon of wind and solar abandonment. During periods of high load demand, energy storage discharge can be used to compensate for any temporary power shortage in the power grid.

### 5.3. Model comparison

This section compares and analyzes the results of energy storage capacity and operating costs obtained by the model in this paper, the deterministic model, and the RO model at a 50% penetration rate of wind and solar energy. Table 3 presents these findings.

**Table 3:** Operating costs for different models.

Result	Proposed model	Deterministic model	Robust model
Electricity purchase cost ($)	9 799.81	9 702.73	9 816.24
Line loss cost ($)	134.63	133.49	138.68
Abandoned Wind Power Rate (%)	0.34%	1.86%	1.31%
Abandoned photovoltaic rate (%)	0.10%	1.25%	0.86%
ESS1(MWh)	0.77	0.74	0.83
ESS2(MWh)	0.63	0.63	0.67

Compared to the deterministic model, the electricity purchase cost is higher, but the wind and light abandonment rate is lower. This encourages the consumption of wind and solar energy. The results suggest that after considering the uncertainty of wind and solar energy, as well as the double-layer uncertainty of scenario probability distribution, configuring energy storage capacity will increase in conservatism. The system will reserve a margin to cope with significant wind and solar uncertainty fluctuations, reducing wind and light abandonment. Additionally, increased participation of ESS in scheduling can improve load matching.

Though this method has a lower configured energy storage capacity than RO, it results in lower electricity purchase costs, line loss costs, and wind and solar abandonment rates. RO often opts for the worst-case scenario, which results in overly conservative outcomes. This method enables a suitable equilibrium between operational efficiency and prudence, allowing configured energy storage capacity to more effectively adjust to the unpredictability of wind and solar output, better aligning with reality.

### 5.4. Algorithm performance analysis

The paper presents the solution of the two-stage distributionally robust model for configuring energy storage capacity using the C&CG algorithm. Table 4 illustrates the results corresponding to each iteration at 50% wind and solar permeability, totaling 201s for the three iterations. The findings indicate that the C&CG algorithm effectively solves the proposed model in this paper since the iteration target value is less than the given solution accuracy after two iterations.

**Table 4:** C&CG algorithm iteration results.

Iterations	Upper bound value/(10^4^ $)	Lower bound value/(10^4^ $)
1	9.7570	9.7313
2	9.7570	9.7570
3	9.7570	9.7570

## 6. Conclusions

This article introduces a two-stage model for configuring energy storage capacity in distribution networks for high-permeability wind and solar power. The model takes a distributionally robust approach based on simulations conducted on an improved version of the IEEE 33-node system. The following conclusions were drawn from the analysis.

1. The suggested approach and procedure can effectively address the configuration of energy storage capacity in high-permeability wind and solar power distribution networks. The proposed energy storage capacity is about 30% of the overall installed capacity of wind and solar power.
2. The configurational outcomes become more conservative because of the unpredictability of wind and solar output and the dual-layer uncertainty of scene probability distribution. Compared to deterministic and robust models, the model presented in this paper attains a favorable balance between economy and conservatism.
3. The research on distributed robust energy storage capacity allocation for high permeability wind and solar power distribution networks in the paper did not emphasize the impact of high permeability on the distribution network. Subsequent research can focus on the impact of high penetration wind and solar energy on distribution networks and analyze their similarities and differences with low penetration rates.

## Supporting information

S1 File (ZIP)
