# Supplementary material for: Research on distributionally robust energy storage capacity allocation for output fluctuations in high permeability wind and solar distribution networks
Source: PLoS One. 2024 Mar 19;19(3):e0299226. doi: 10.1371/journal.pone.0299226 (PMC10950218; doi:10.1371/journal.pone.0299226)
Supplement: S1 File — (ZIP) [file pone.0299226.s001.zip › Model parameter..docx]

| **Parameters** | **Numerical value** | **Parameters** | **Numerical value** |
| --- | --- | --- | --- |
| ESS Life Cycle (Years) | 20 | Maximum ESS charging and discharging power (MW) | 0.3 |
| ESS discount rate | 0.08 | ESS charging efficiency | 0.938 1 |
| ESS unit installation price ($/MW) | 240 000 | ESS discharge efficiency | 0.938 1 |
| ESS Aging Cost ($/MWh) | 20 | Total active load (MW) | 3.715 |
| PV Abandoned photovoltaic Price  ($/MWh) | 50 | Total reactive load (Mvar) | 2.3 |
| WT Abandoned Wind Price ($/MWh) | 50 | Total simulation period (h) | 24 |
| Network loss price ($/MWh) | 50 | Line capacity (MW) | 6 |
| Upper voltage limit (p.u.) | 1.06^2^ | Upper limit of WT power factor | 0.9 |
| Lower voltage limit (p.u.) | 0.94^2^ | Number of historical data | 5 000 |
| Substation node | 33 | 1/∞-norm corresponds to uncertainty probability confidence | 0.99 |
